# Supplementary material for: IsoAnalytics: a single-cell proteomics web server
Source: Bioinform Adv. 2023 Jun 21;3(1):vbad077. doi: 10.1093/bioadv/vbad077 (PMC10290237; doi:10.1093/bioadv/vbad077)
Supplement: vbad077_Supplementary_Data [file vbad077_supplementary_data.docx]

IsoAnalytics: A Single-cell Proteomics Web Server

Suzette N. Palmer^1,2,3^, Andrew Y. Koh^1,4,5,*^ and Xiaowei Zhan^3,5,*^

^1^Department of Pediatrics, Division of Hematology/Oncology, The University of Texas Southwestern Medical Center, Dallas, TX 75390 ^2^Department of Biomedical Engineering, The University of Texas Southwestern Medical Center, Dallas, TX 75390 ^3^Peter O’Donnell Jr. School of Public Health, Quantitative Biomedical Research Center, The University of Texas Southwestern Medical Center, Dallas, TX 75390 ^4^Department of Microbiology, Division of Hematology/Oncology, The University of Texas Southwestern Medical Center, Dallas TX 75390 ^5^Harold C. Simmons Comprehensive Cancer Center, The University of Texas Southwestern Medical Center, Dallas TX 75390

*To whom correspondence should be addressed.

# **Supplemental Material**

## Sections

## [Tutorial for IsoAnalytics](#s1)

- 1. [Upload Isoplexis Data](#upload)
  2. [Hierarchical Clustering Analysis](#hc)
  3. [Dimensionality Reduction Analysis](#dr)
  4. [Polyfunctionality](#p)
  5. [Statistics and Distribution](#dis)

## [FAQs](#s22)

Section 1: Tutorial for IsoAnalytics

1. Login to the following Webserver: <https://cdc.biohpc.swmed.edu/isoplexis/>. The user will be directed to the “Overview” tab, which contains descriptions of the analysis and visualization pages, details of the Isoplexis single cell secretome assays and contact information.
   1. For inquiries, please contact Xiaowei Zhan, Ph.D., via email at [Xiaowei.Zhan@utsouthwestern.edu](mailto:Xiaowei.Zhan@utsouthwestern.edu).
   2. For issues, please create a new issue through <https://github.com/suziepalmer10/Isoplexis_Data_Analysis/issues>.


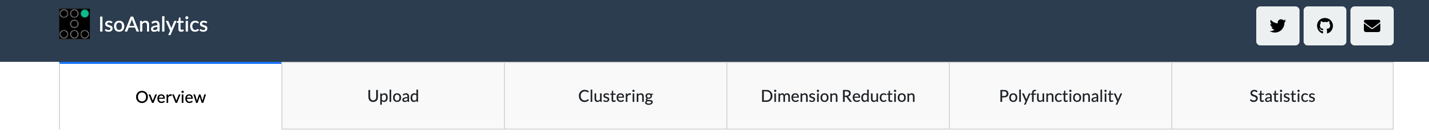


1. To begin the analysis, select the Upload tab. Follow all five steps listed below.
   1. Step 1: Upload Isoplexis single cell data, which can be formatted as a CSV or Excel (.xlsx). Once the file is successfully uploaded, the name of the file and the timestamp of the file will appear below the instructions for step 1. We have included an option to directly upload an example file, which will be directly uploaded when the user clicks the button “Upload Example”.


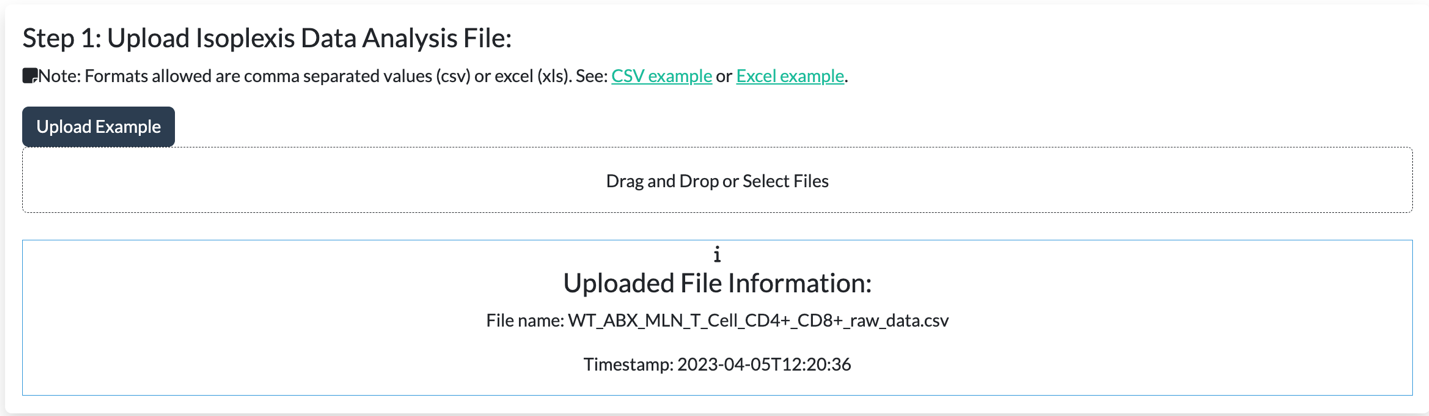


- 1. Step 2: Select the assay/panel used for the Isoplexis data. This will continue to be updated as Isoplexis assays are modified and/or developed. Current Isoplexis single cell assays are shown below.


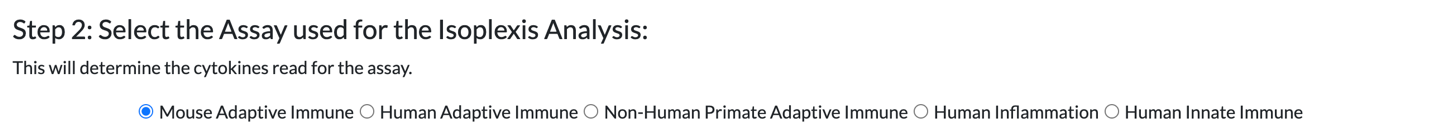


- 1. Step 3: Select the conditions that will be analyzed. A drop-down menu displays the conditions that can be selected and analyzed. The order in which the conditions are selected will be the same order displayed for the visualizations in the data analysis tabs. Once the order is selected, press the “Reorder Data” button below. Note: if the user decides to select different conditions to analyze, this step, along with steps 4, 5 and 6 must be repeated.


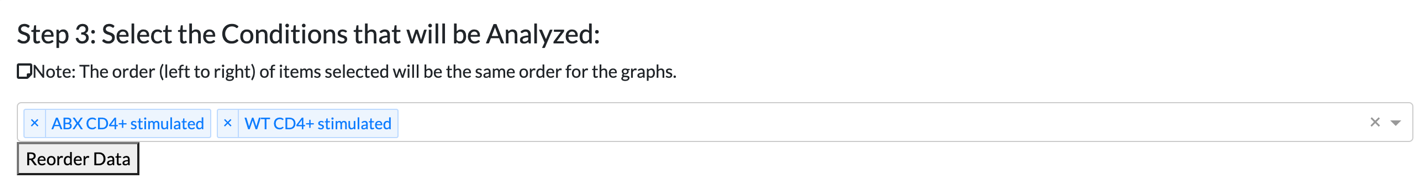


- 1. Step 4: User options to scale, normalize and filter the data. The user has 4 options of normalization and scaling, which include “None”, “Log Scale”, “Normalize by cytokine” and “Log scale and Normalize by Cytokine”, The option, “None” will not perform any scaling or normalization. The “Log Scale” option will perform a log10 transformation on the data. The option “Normalize by Cytokine” will normalize the data across each cytokine. The option, “Log scale and Normalize by Cytokine” will first log 10 transform the data and then normalize the data across each cytokine. The user also has the option to remove cells that do not express any cytokines. This option can significantly reduce the dataset and associated sparsity, since cells with no cytokine expression are removed.


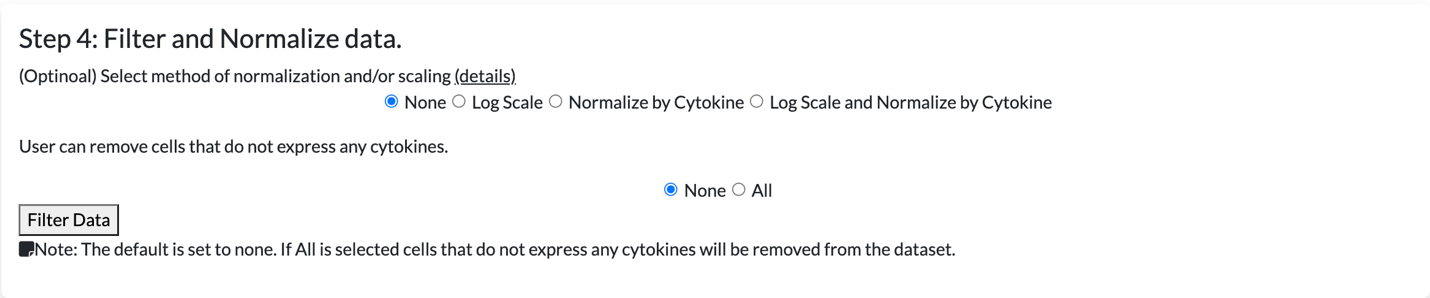


- 1. Step 5: Select the “Analyze Isoplexis Data” button. Please ensure the number of cells and cytokines are correct before proceeding.


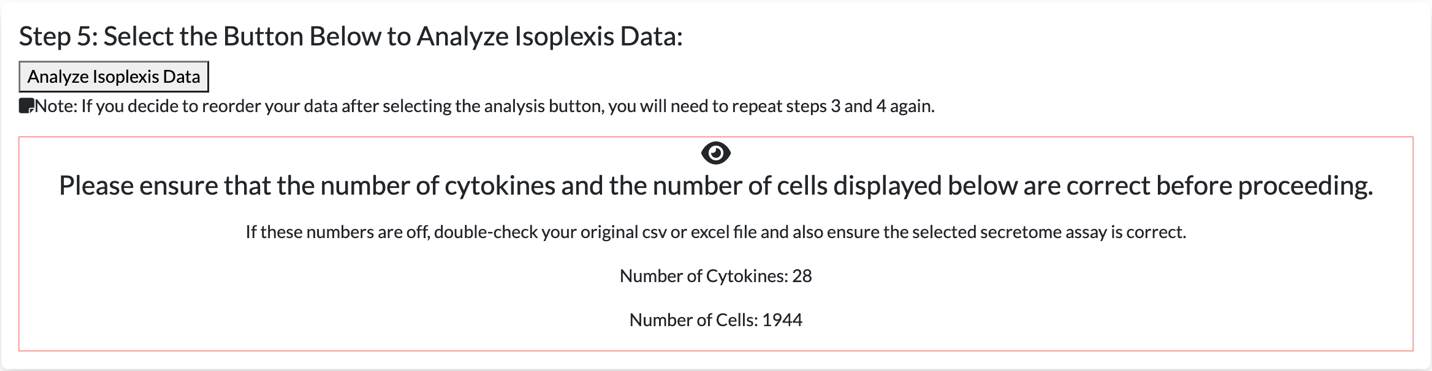


- 1. Step 6: Individual Cytokine Analysis. Select one cytokine for the individual cytokine analysis. Note: if the user wishes to select another cytokine, step 6 must be repeated.


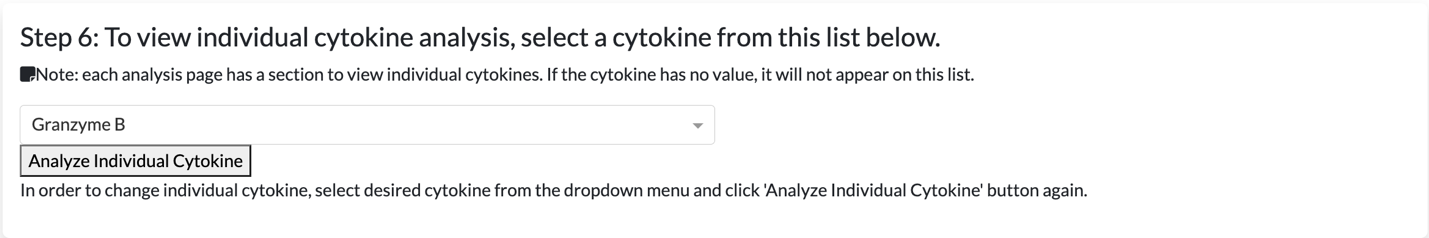


1. Hierarchical Clustering visualized using a clustered heatmap.
   1.
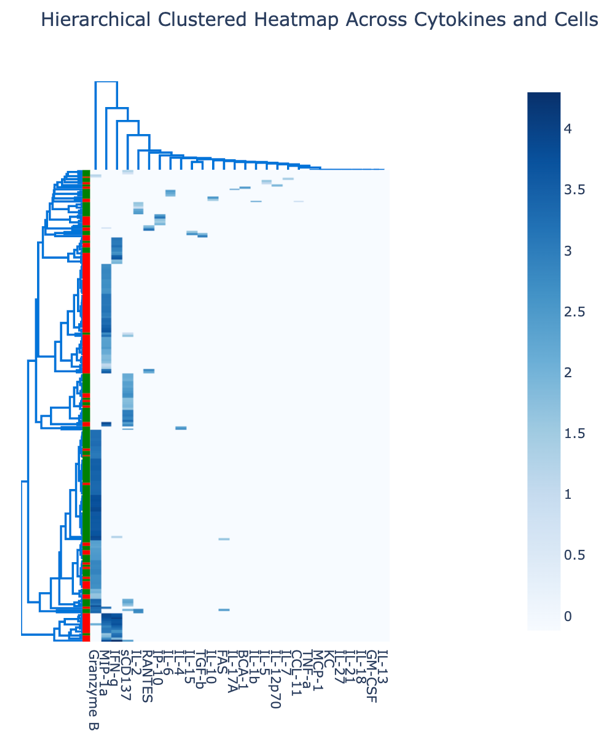
Goal: The goal of this analysis is to identify population of cells that might be important for immune function. This analysis allows the user to more easily visualize clusters of cells that may be expressing similar cytokine patterns, such as the red condition cluster outlined in purple. Additionally, by visualizing these clusters, the user is also able to identify polyfunctional cells and similar expression patterns, which can give insight into the kind of immune responses that are occurring and that are different/similar amongst the treatment conditions.
   2. For the clustering analysis, all or individual conditions can be selected. The visualizations will automatically update after selection.


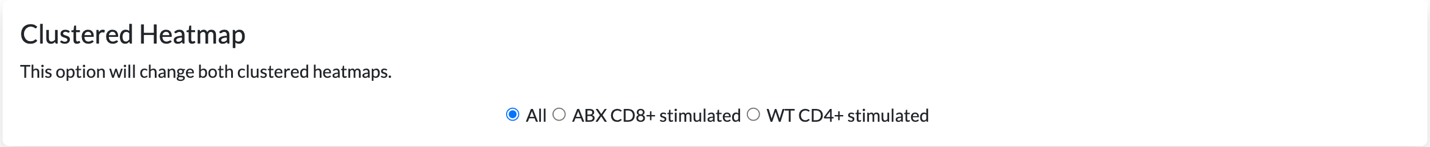


- 1. The user can select whether to cluster by cytokines or by cells.


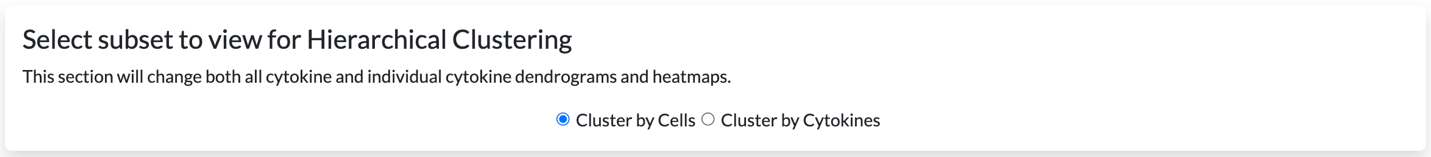


- 1. Clustered heatmap for all cytokines. Advanced features of the visualizations include zoom, pan, auto scale, and reset axes. Visualizations can be downloaded as a png image. Hover text is also implemented in the heatmap.


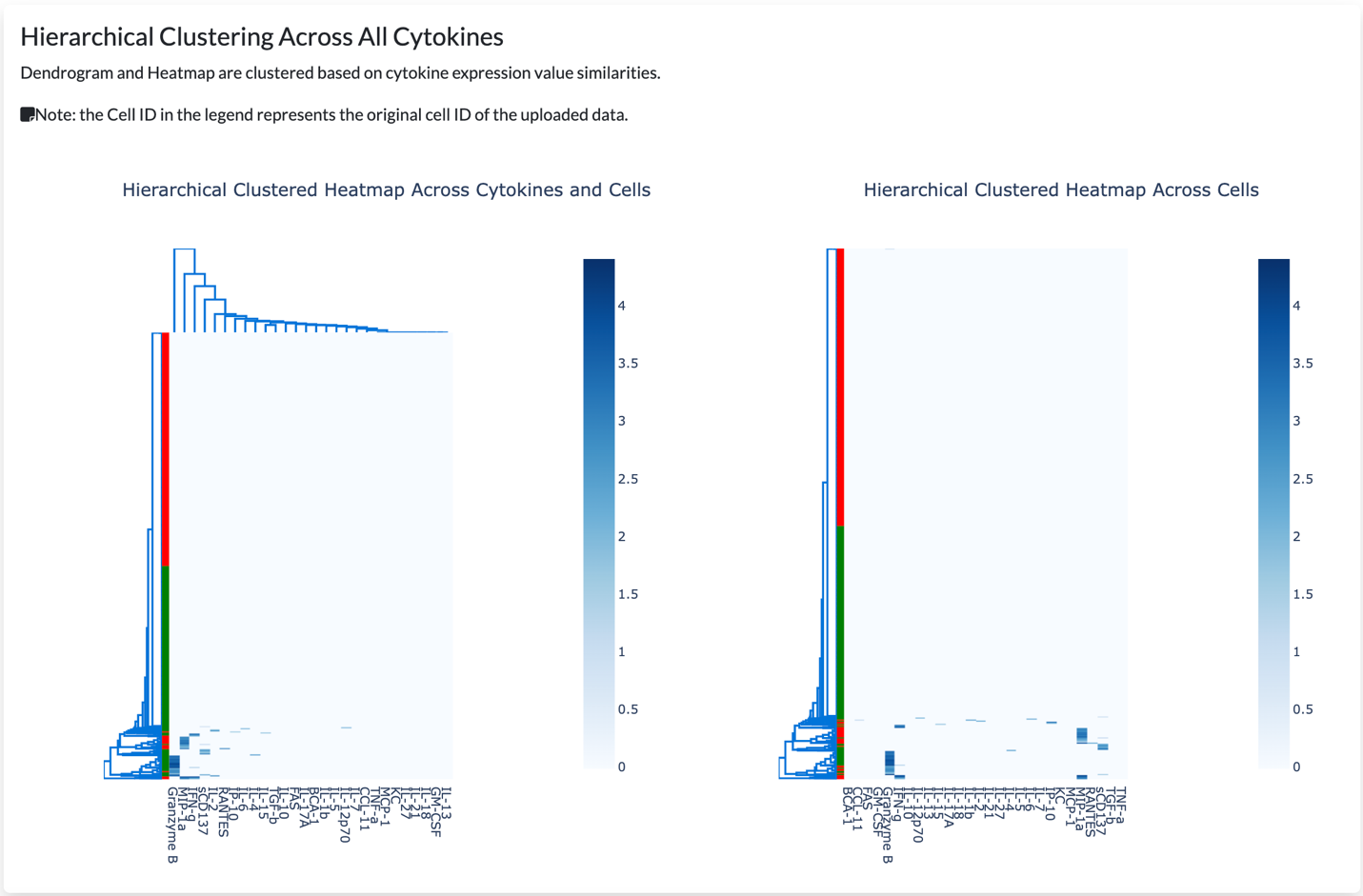


Example Features (described above):

Zoom Hover text
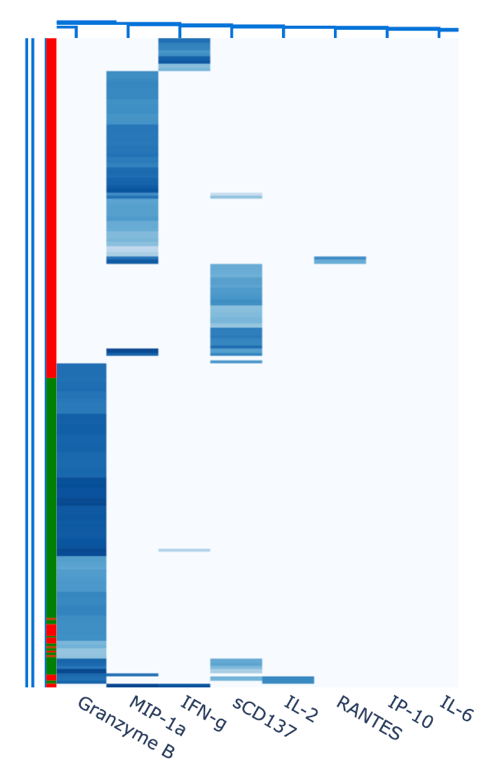

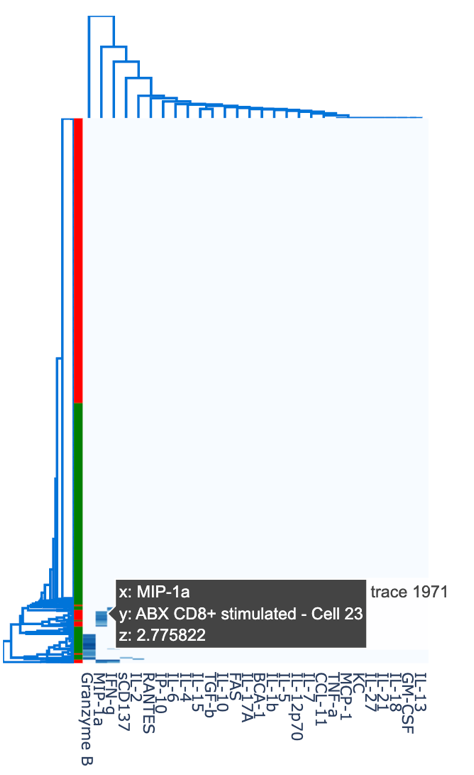


- 1. Dendrogram for cells with cytokine present. This analysis is based on the individual cytokine selected [(2f)](#e2). Only cells that contain the cytokine are used for clustering. The same features outlined above [(3b)](#b3) are also applicable for these visualizations.


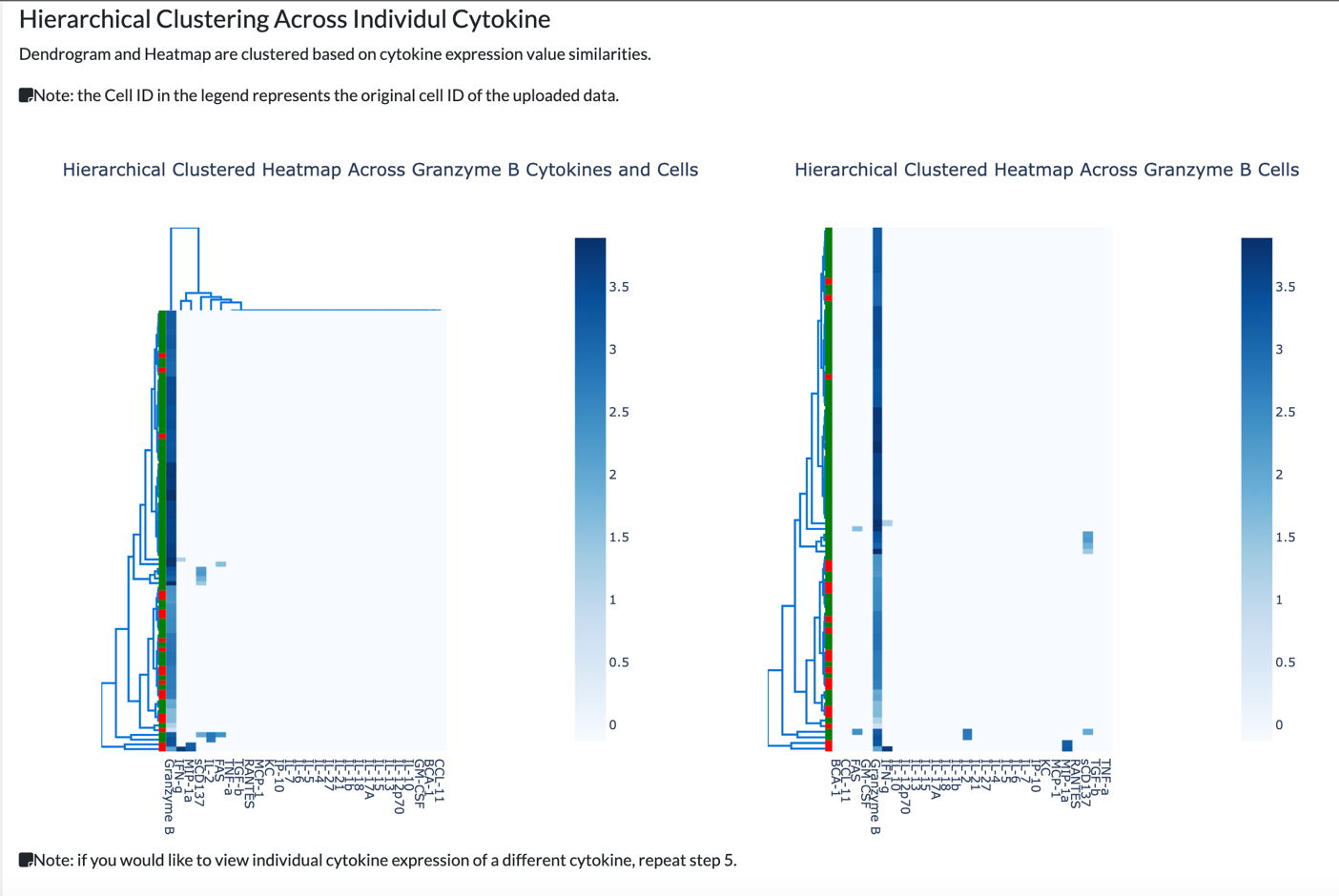


1.
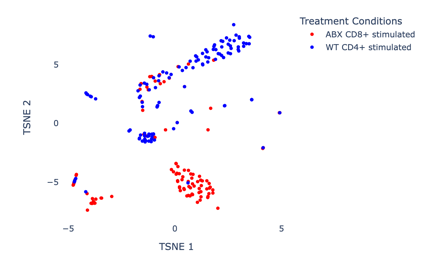
PCA and TSNE Dimensionality Reduction
   1. Goal: The goal of these analyses are to allow the user to visualize whether the treatment condition groups separate as potentially expected by using dimensionality reduction techniques, PCA and TSNE. A TSNE example is shown to the left, where the WT CD4+ cells (blue) generally cluster together and the ABX CD8+ cells (red) generally cluster together. Note: This analysis works better for filtered data (removing cells with 0 cytokine expression).


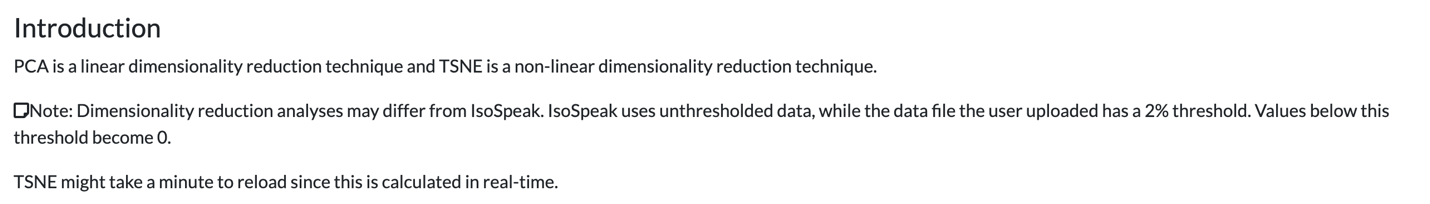


- 1. User options for PCA and TSNE include scaling and 2D/3D visualization


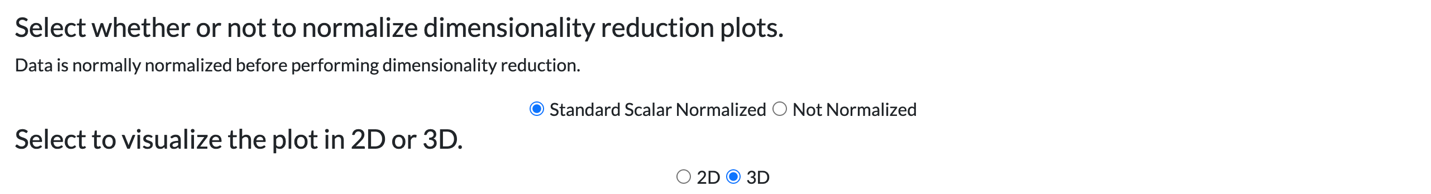


PCA Examples:

3D PCA 2D PCA


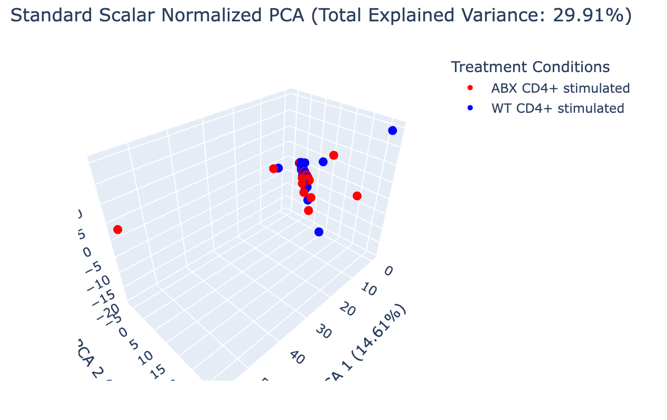

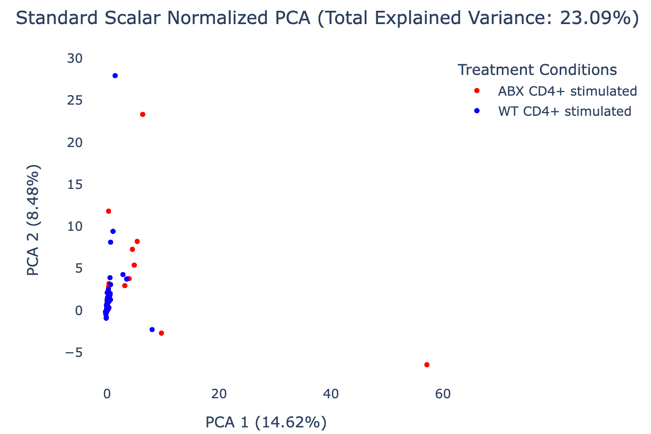


- 1. For TSNE, the user has the option to optimize two hyperparameters – perplexity of nearest neighbors and number of iterations.

TSNE Examples:

3D TSNE 2D TSNE


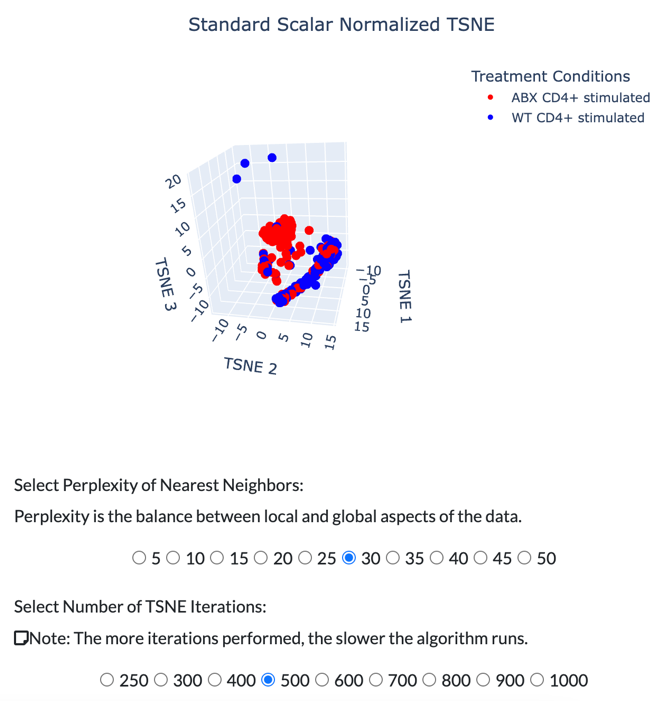

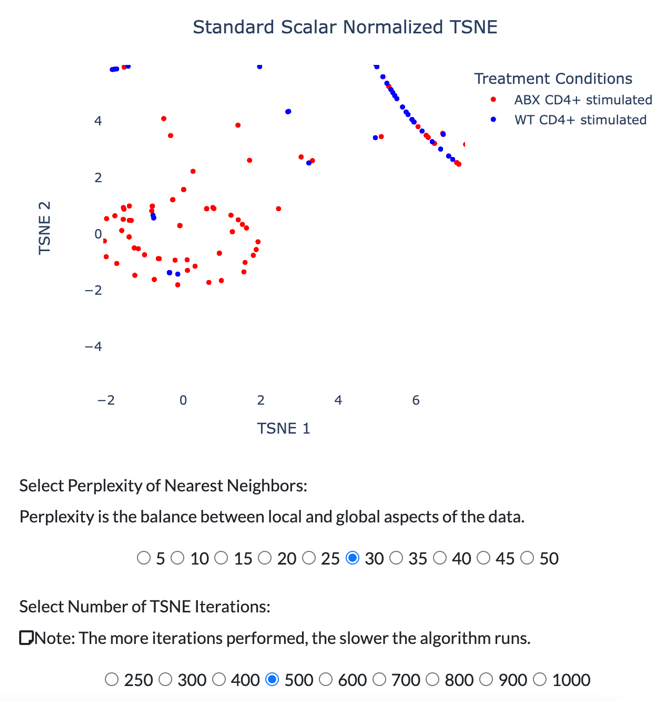


- 1. Advanced features of the visualizations include zoom, pan, auto scale, hover text, and reset axes. Visualizations can be downloaded as a PNG image. The user can also select individual treatment conditions to view.

Example features (described above):

Specific condition selected to view Hover text


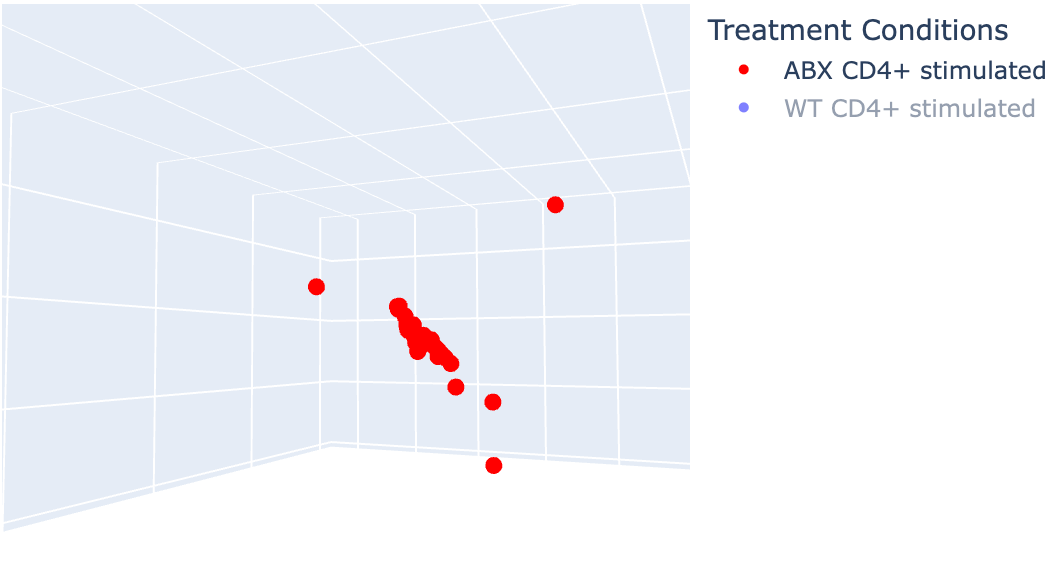

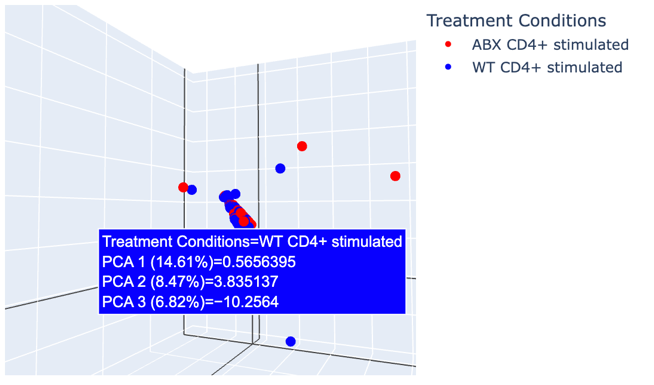


1. Polyfunctionality
   1. Goal: The goal of this analysis is to calculate and visualize the number of polyfunctional cells in each treatment condition. The quantity of polyfunctional cells that differ between treatment conditions could be indicative of increased or decreased immune response (i.e., the greater number of polyfunctional cells, the more active the immune response). Additionally, individual cells that express more cytokine are more protective against potential pathogens and/or disease (Foley, 2012; DOI:[10.1126/scisignal.2002929](https://doi.org/10.1126/scisignal.2002929)). The dominant functional groups allow the user to gauge which functional group (indicative of immune function) is most prevalent in each of the treatment conditions. If the immune response is different between treatment conditions, this may explain why the phenotype and cytokine expression differs between the groups.
   2. The number of polyfunctional cells for the assay is displayed at the top left corner of the “Polyfunctionality” tab.


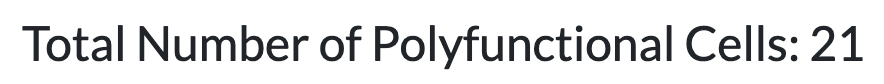


- 1. The stacked bar graph on the left displays the percent of cytokines secreting for each condition, which calculates the proportion of cells that express two or more proteins. The user can download these values as a CSV file.


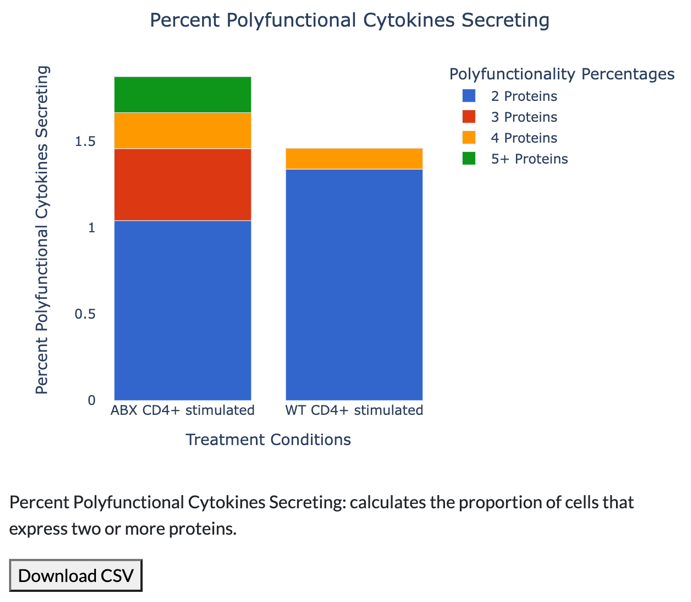


CSV Example Output


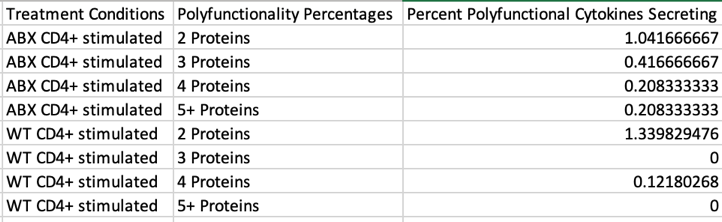


- 1. The stacked bar graph on the right displays absolute abundance or proportion of dominant functional groups for the secreting cytokines as classified by Isoplexis. These values can be exported as a CSV file.


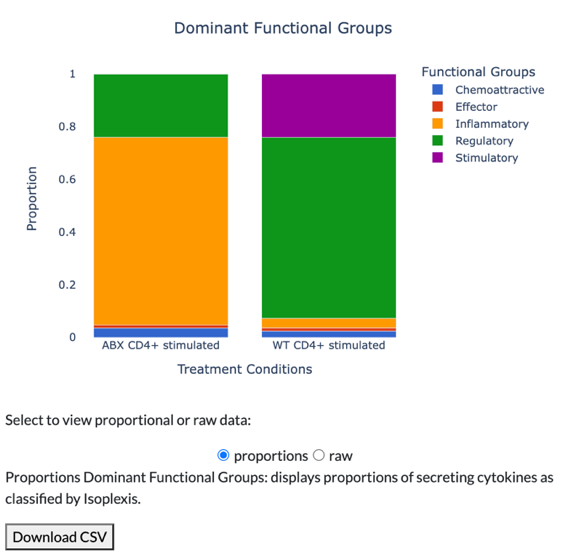


- 1. Advanced features of the visualizations include zoom, pan, auto scale, hover text, and reset axes. Visualizations can be downloaded as a png image. The user can also select individual treatment conditions to view.

Example features (described above):

Specific condition selected to view Hover text


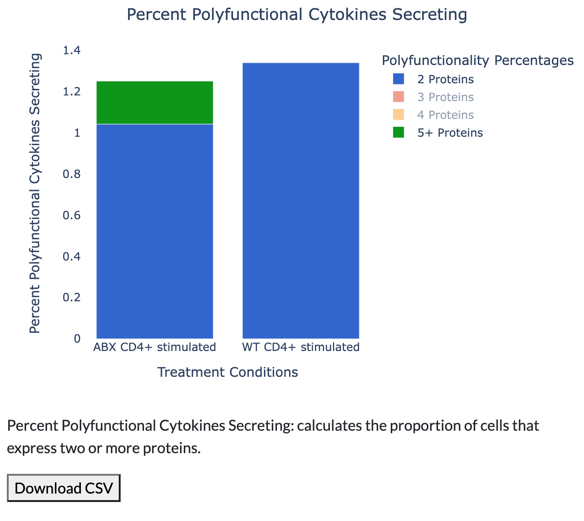

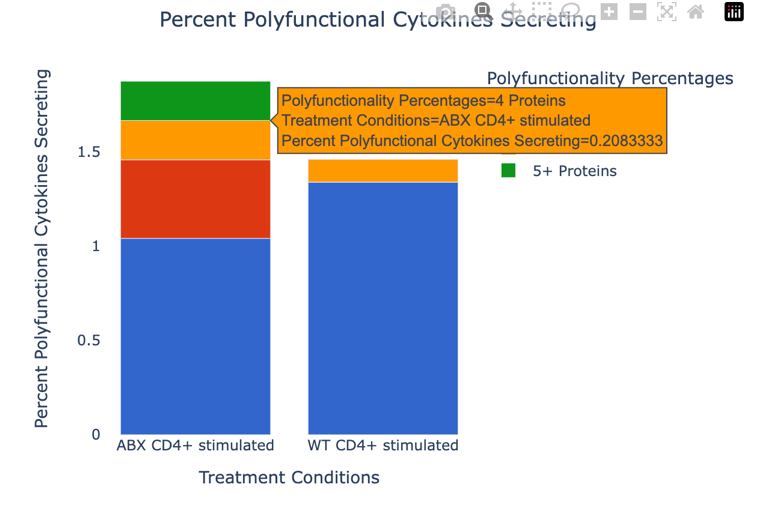


1.
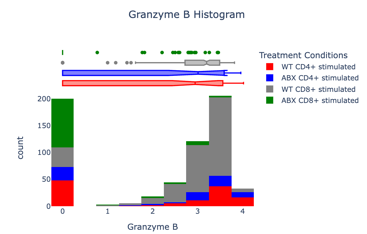
Distributions and Statistics
   1. Goal: The goal of this page is for the user to view distribution and carry out statistical tests of their data for each cytokine across treatment conditions. For example, by visualizing the histogram to the right, we can clearly see that CD8+ stimulated cell conditions have greater cytokine expression than CD4+ stimulated cell conditions, regardless of WT or ABX treatment conditions. Additionally, statistical tests can easily be performed by the user and these findings can be incorporated into their future manuscript.
   2. Percent cytokines secreting (non-zero proportions) across all cytokines.


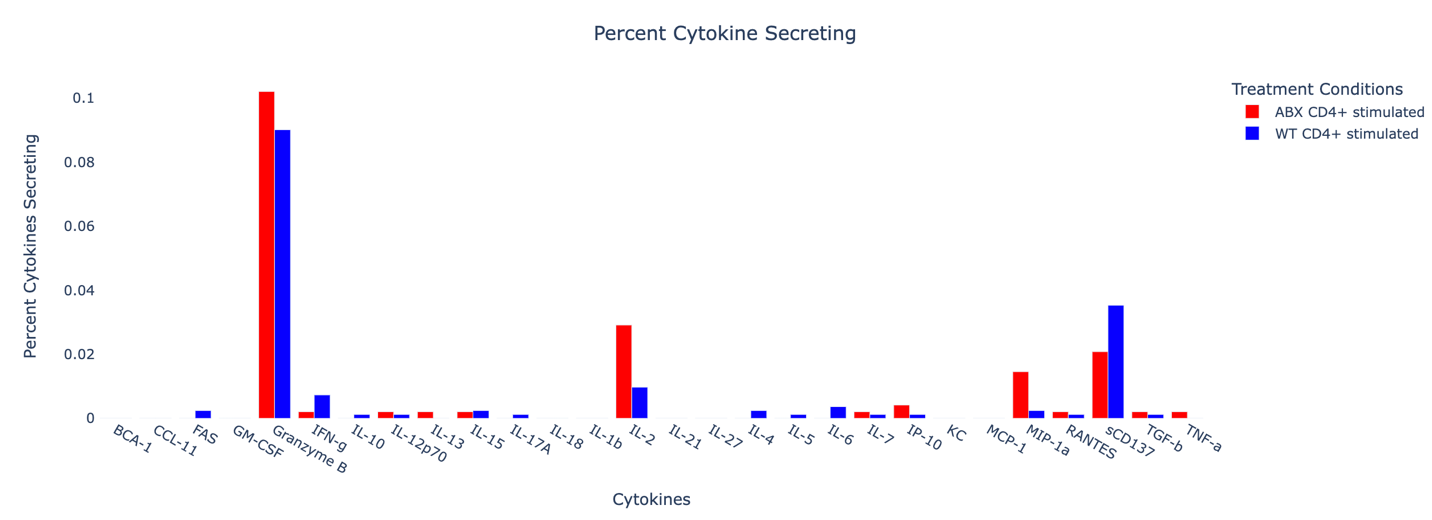


- 1. Individual cytokine statistical summary displays statistics for the selected cytokine [(2e)](#e2). The user can select all treatment conditions or an individual treatment condition.


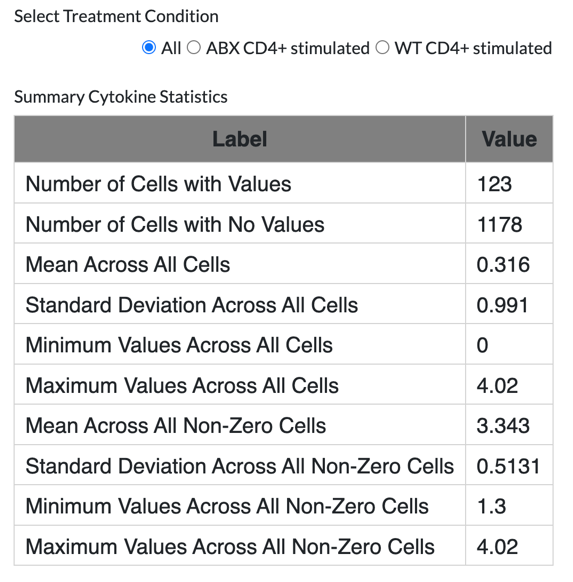


- 1. Histogram of the selected cytokine [(2e)](#e2) and user choice of box plot, violin plot or rug plot displayed above. Additionally, the user can change the bin sizes for the histogram.


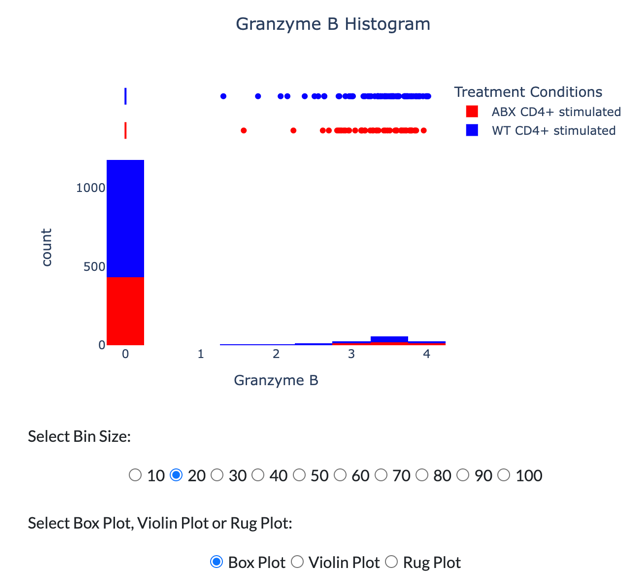


- 1. Statistical tests include non-zero proportion and Kolmogorov-Smirnov. The user can select which conditions to compare for these tests, and the z-statistic and p-value are displayed below.


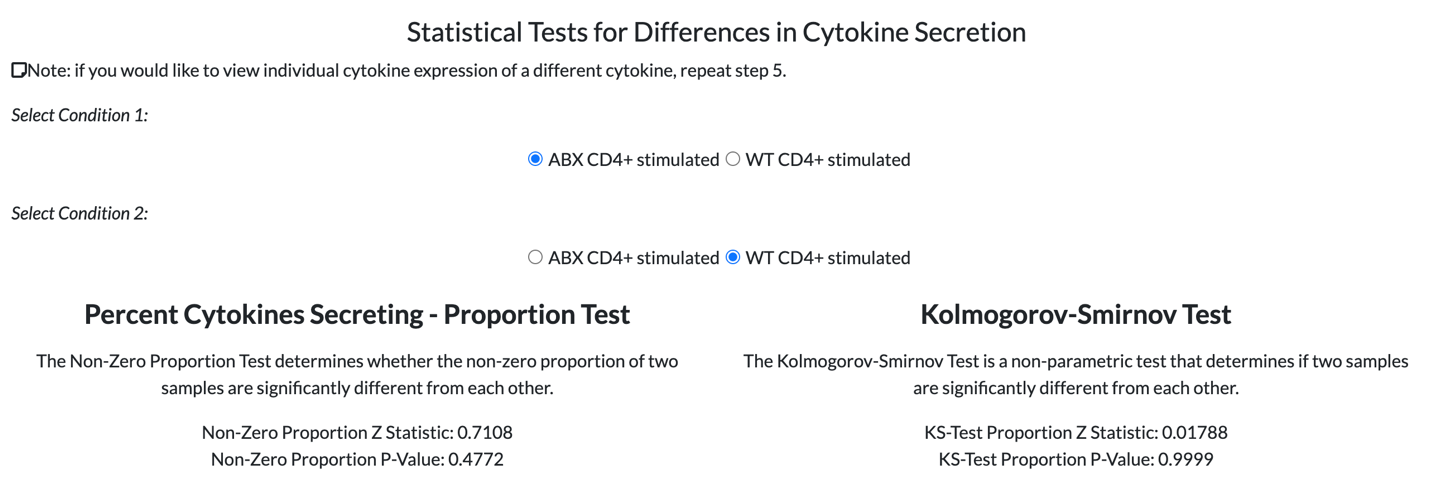


- 1. Individual cytokine non-zero proportion bar graph is displayed on the bottom left of the “Distribution and Statistics” tab.


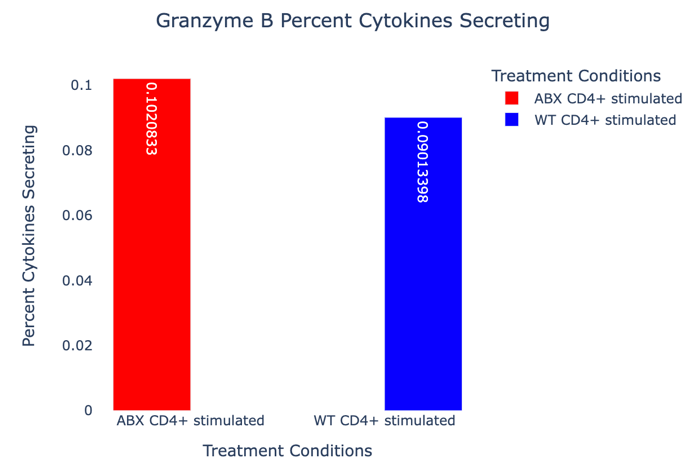


- 1. Individual cytokine density plot is displayed on the bottom right of the “Distribution and Statistics” tab.


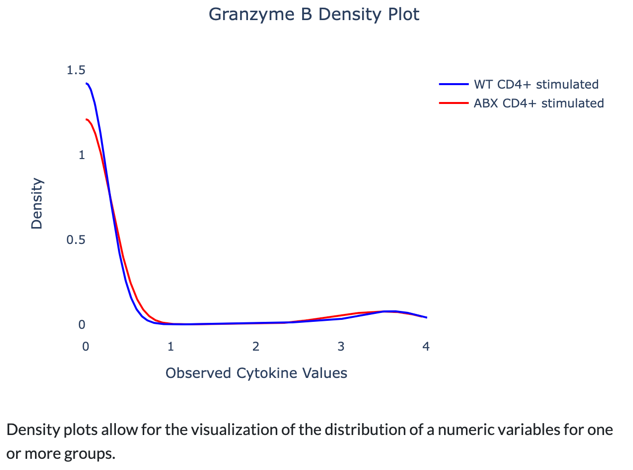


- 1. Advanced features of the visualizations include zoom, pan, auto scale, hover text, and reset axes. Visualizations can be downloaded as a png image. The user can also select individual treatment conditions to view.

Section 2: FAQ

1. How often is IsoAnalytics updated?
   1. We will check for new Isoplexis single-cell assay modifications and developments monthly to ensure that our web server is keeping up with Isoplexis technology.
2. Suggestions on other methods and visualizations that you (an IsoAnalytics user) would like to see?
   1. Please reach out to Xiaowei Zhan using the email [Xiaowei.Zhan@utsouthwestern.edu](mailto:Xiaowei.Zhan@utsouthwestern.edu). We are very open to feedback, and we would likely incorporate these suggested features/analysis/methods into our web server.
3. Data is not loading? What should you do?
   1. Check the file. Importantly, this website uses the columns “Donor”, “Cell Subset” and “Stimulation” to categorize the treatment conditions. Ensure that the file has these metadata columns.


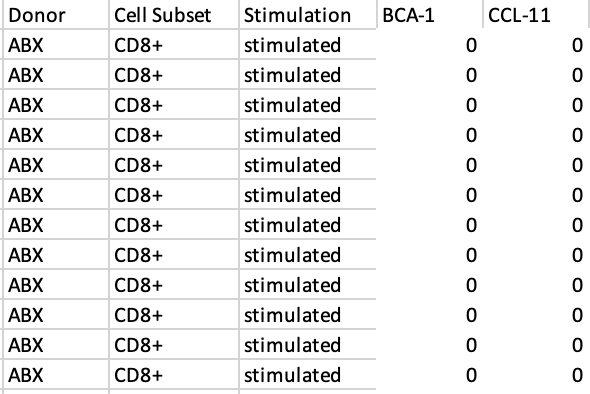


- 1. Check that the correct assay on the “Upload” tab has been selected. Currently there are five single-cell assays (listed below). This website relies on the presence of the correct list of cytokines used for each assay.


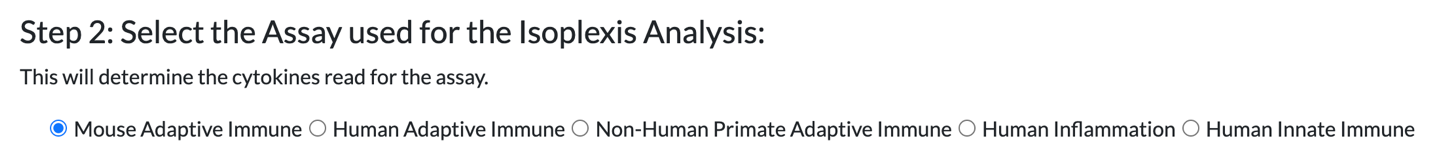


- 1. Still not working? Please contact Xiaowei Zhan using the email [Xiaowei.Zhan@utsouthwestern.edu](mailto:Xiaowei.Zhan@utsouthwestern.edu). We will help you troubleshoot.

1. Notice a bug or glitch?
   1. For issues, please create a new issue through <https://github.com/suziepalmer10/Isoplexis_Data_Analysis/issues>.
